# Recognition of emotions in German laughter across cultures

**DOI:** 10.1038/s41598-024-53646-4

**Published:** 2024-02-06

**Authors:** Diana P. Szameitat, André J. Szameitat

**Affiliations:** Centre for Cognitive and Clinical Neuroscience, Division of Psychology, Department of Life Sciences, College of Health, Medicine and Life Sciences, Brunel University London, Kingston Lane, Uxbridge, UB8 3PH UK

**Keywords:** Psychology, Human behaviour

## Abstract

Laughter conveys a wide range of information relevant for social interaction. In previous research we have shown that laughter can convey information about the sender’s emotional state, however other research did not find such an effect. This paper aims to replicate our previous study using participant samples of diverse cultural backgrounds. 161 participants from Poland, the UK, India, Hong Kong, and other countries classified 121 spontaneously emitted German laughter sounds according to the laughter type, i.e., joyful, schadenfreude, and tickling laughter. Results showed that all participant groups classified the laughter sounds above chance level, and that there is a slight ingroup advantage for Western listeners. This suggests that classification of laughter according to the sender’s emotional state is possible across different cultures, and that there might be a small advantage for classifying laughter of close cultural proximity.

## Introduction

Laughter is a nonverbal signal that plays an important role in social interaction and affects the emotional state of laughers^1^ and listeners^2^ alike. In recent years, the function of laughter as a communicative tool has been extensively investigated. However, empirical work on how people perceive laughter is still limited^3^.

One of the main questions is what kind of information is communicated from the person who laughs (sender) to the listener (receiver) by means of the mere acoustic laughter signal, i.e., without information about facial expressions or the context in which the laughter is uttered. For example, research has shown that laughter can communicate information regarding affiliation and group structure, i.e., listening to laughter listeners can infer the social status of the sender^4^ and whether the sender laughs among friends versus strangers^5^ or among friends versus their lover^6^. Moreover, listeners are able to perceive whether laughter is uttered spontaneously or voluntarily (also called posed or faked laughter)^7–9^.

In addition, there is accumulating evidence that laughter, like facial expressions^10^ and emotional prosody in speech^11^, can communicate information about the affective state of the laugher. For example, in previous studies we were able to show that listeners can distinguish between spontaneous joy laughter, schadenfreude laughter (laughing about the misfortune of someone else), and tickling laughter^12^ and between joy, schadenfreude, taunting and tickling laughter generated by professional actors^13^. In line, Wood and colleagues showed that the acoustic variation of laughter taken from a commercial sound database (acted laugher) is linked to the perception of reward, affiliation and dominance^14^, and that spontaneously emitted laughs (natural laugher) emitted in different social contexts show distinct acoustic profiles^15^. Furthermore, spontaneous laughter produced in conversations differs acoustically from mirthful laughter^16^.

However, the general notion that the mere acoustical signal of spontaneous laughter can communicate the affective state of the sender without any contextual information has been questioned. For example, Scott^17^ suggested that it is mostly the situational context of the listener (receiver) which determines the perception of the laughter sound, e.g. whether one feels being laughed at. This suggestion is in line with empirical findings by Rychlowska et al.^18^ who found that listeners were able to classify natural laughter that was uttered in situations evoking amusement, embarrassment, and schadenfreude only with contextual information, but not based on the acoustical signal alone. In line with this, Suarez and colleagues suggested that laughter carries distinct emotions, but that contextual information is necessary to distinguish the affective state of the laugher^19^. Therefore, the first aim of the current paper was to provide further findings to the currently contradictory empirical evidence regarding the communication of the affective state in laughter^12,18^. For this, we conducted a study similar to Szameitat et al.^12^, but in a much larger and more diverse sample. We predicted that the affective state of the sender can be recognised above chance level by listeners of diverse cultural backgrounds, which would provide further evidence that the mere acoustical laughter signal can communicate the affective state of the sender without any situational context.

The second aim of the current paper was to gather first evidence whether the affective state of the sender can also be communicated across cultures. The influence of culture on emotional communication is a key question in all forms of emotional communication, such as facial expressions^20^ and emotional prosody in speech^21^. The overall finding is that basic emotions, such as fear, happiness, sadness, anger, disgust, and surprise can be recognised across cultures, with an in-group advantage for listeners of close cultural proximity^22–24^.

Recent articles showed that laughter expression is also shaped by cultural influences^25–27^. While laughter among other non-verbal vocalizations (e.g. laughter for amusement, sobbing for sadness, screaming for fear) can be recognized across cultures, there was an in-group advantage for cultures of close proximity, indicating the existence of cultural influences on the perception of non-verbal emotional vocalizations^27^. With respect to laughter, Kamiloğlu et al.^25^ reported that laughter sounds more positive when produced by listeners close to the sender’s own culture. For nonverbal vocalizations it has been shown that, even though it is unclear whether listeners are generally able to identify the cultural background of the sender on the basis of the nonverbal vocalisation^25,26,28^, there seems to be an improved emotion recognition (for triumph, relief, amusement) of in-group signals^28^. A similar cultural in-group advantage might also be expected for the classification of laughter sounds according to the affective state of the laugher (sender).

Taken together, while there are indications that laughter in general is a cross-cultural phenomenon which may be influenced by the cultural background, studies investigating whether the affective state of the laugher (sender) is communicated across cultures are missing. To test this in the current paper, we used a set of spontaneously emitted joy, schadenfreude and tickling laughter stimuli voiced by German speakers, as created and used in Szameitat et al.^12^, and tested how well the different types of German laughter were recognised by listeners from the UK, Poland, India, Hongkong, and a mixed group of non-German listeners not fitting any of the other categories (“Other”). We predicted, first, that listeners from other cultures are able to classify spontaneous German laughter according to the sender’s affective state above chance level and, second, that classification rates are higher for listeners with a close cultural proximity to the German culture.

## Methods

### Participants

The current study was run at Brunel University London and is based on the final year dissertations of three BSc students and one MSc student with cultural backgrounds in Poland, India, Hong Kong, and China. All studies were approved by the Department of Life Sciences Ethics committee, Brunel University London, UK, were performed in accordance with relevant guidelines and/or regulations, and were performed in accordance with the Declaration of Helsinki. BSc students tested 95 participants recruited via opportunistic sampling using social media adverts in the UK and other countries. These 95 participants were unpaid or received course credit for participation. The MSc student tested 66 participants recruited via Testable Minds (https://minds.testable.org/) and those participants (29 India, 20 Other, 16 UK, 1 Polish) were paid US$4 for participation. Overall, 161 participants (83 females, 78 males) were recruited (mean age 30.01 years, see Table 1 for details).

**Table 1:** Demographic data.

	Overall	Poland	UK	India	Hong Kong	Other
N Total	161	20	37	45	25	34
N Females	83	15	23	15	15	15
N Males	78	5	14	30	10	19
Mean Age	30.01	33.5	26.92	26.84	42.92	26.03
Age SD	13.37	12.38	10.40	7.86	20.62	9.89
Age Min	18	20	18	18	18	19
Age Max	81	67	60	51	81	65

To determine the cultural background of the participants, we asked the places of birth of the participants and their parents, and where participants and their parents have lived most of their lives. Since this was not meant to be a primarily cross-cultural study, we were rather lenient during the recruitment process and based the classification into cultural groups on the question with which culture the participant identified most with (this was an open question, not a choice among given cultures/countries). Table 2 provides an overview of the cultural backgrounds of the groups. There were a further 34 participants with a variety of cultural backgrounds (in order of frequency (N): Arab (3), Philippines (3), Bangladesh (2), Italy (2), Somalia (2), Sri Lanka (2), United States (2), and one participant each identifying most with Afghanistan, Algeria, Brazil, Colombia, Croatia, Greece, Iran, Latin America, Lithuania, Malaysia, Pakistan, Serbia, South Africa, Spain, Turkey, and 3 participants with unclear cultural background). The latter mixed group was collated into a group called “Other”.

**Table 2:** Cultural background of the different groups. Participants were categorised by the culture they identified most with. There was a further group “Other” with participants from various backgrounds, see text for details.

Group	Answer	P—PoB	P—Lived	M—PoB	M—Lived	F—PoB	F—Lived
Poland	*Poland*	20	20	20	19	19	19
N = 20	*Not Poland*	0	0	0	1	1	1
UK	*UK*	36	37	30	32	28	35
N = 37	*Not UK*	1	0	7	5	9	2
India	*India*	44	44	45	44	44	43
N = 45	*Not India*	1	1	0	1	1	2
Hong Kong	*Hong Kong*	24	21	20	23	20	22
N = 25	*Not Hong K*	1	3	5	2	5	3

P/M/F—PoB = place of birth participant/mother/father. P/M/F—Lived = where did they live most of their lives (participant/mother/father). Example: In the Hong Kong group, all 25 participants identified most with the Hong Kong culture, although one of them was not born in Hong Kong, and three of them did not live most of their live in Hong Kong, and five of them had mothers not born in Hong Kong, and so forth. Note that most “Not Hong Kong” answers in the Hong Kong group where China.

### Stimulus material

We used a set of 121 spontaneous laughter stimuli generated by 39 German speakers as used and described in Experiment 2 in Szameitat et al.^12^. Joyful and schadenfreude laughter was generated by groups of friends (speakers/senders) watching funny video clips together. The video clips were chosen to elicit various emotional responses, e.g. clips from positive funny situations (e.g. a newsreader misreading and laughing at herself, a baby laughing funnily, etc.), and clips which were meaner in nature (e.g. clips of nasty pranks, such as letting a friend step into mouse traps). After watching the video clips, participants were questioned what emotion they felt during each individual video clip, e.g. joy, embarrassment, cuteness, pity, schadenfreude, taunt, or any other emotion. Tickling laughter was produced by the same groups of friends tickling each other. This resulted in a set of 381 laughter stimuli. These stimuli have then been categorised by German participants (listeners/receivers) in Experiment 1 in Szameitat et al.^12^, and based on those results a sub-set of 121 stimuli was generated, in which each individual laughter stimulus had a correct classification rate of at least 50%. The resulting stimulus set consisted of 121 laughter sequences (50 joy, 42 tickle, 29 schadenfreude, 1–4 per laughter type and sender, 19 male and 20 female senders) with an average classification rate of 56.3% (tickle 52.8%, joy 54.5%, schadenfreude 63.4%). For more methodological details on the stimulus material please refer to Szameitat et al.^12^. Please note that it is not possible to directly compare the results from Szameitat et al.^12^ with the current study due to some methodological changes (e.g. the number of possible answer categories was different).

### Procedure

The study was conducted online using PsyToolKit^29,30^. After providing informed consent, participants were instructed, and then practiced the classification task with 12 laughter sequences which were not part of the main stimulus set. Participants were played a laughter sequence and were asked to indicate whether they thought the laughter sounded most like joy, schadenfreude (the concept was explained to participants), or tickling. To give the answer, participants had to choose one of three answer boxes that were presented horizontally on the screen by mouse click. Participants were asked to first listen to the full laughter sequence before giving their answer. They were able to listen to the same sequence as many times as they wished before giving an answer. The experimental software did not record how often participants made use of this option, but incidental observations of in-person testing in other studies suggest that participants very rarely listen more than once to a stimulus. Once an answer box had been selected, the next laughter was played. The 121 stimuli were split into four blocks of 30–31 stimuli per block, with the option to have a break between blocks. The order of stimuli was randomised across participants. There was no time pressure during the experiment, and the average total duration was about 20 min.

### Statistical analysis

For all statistical tests Wagner’s^31^ unbiased hit rate for correct classification (H_u_) and Wagner’s proportion correct (P_c_) were calculated for each participant and laughter type individually. The comparison of H_u_ with P_c_ accounts for false alarms, uneven stimulus distributions, and response biases^31^. For convenience, for illustration in tables and figures we report hit rates (percent correct).

## Results

### Descriptive statistics

**Figure 1:**
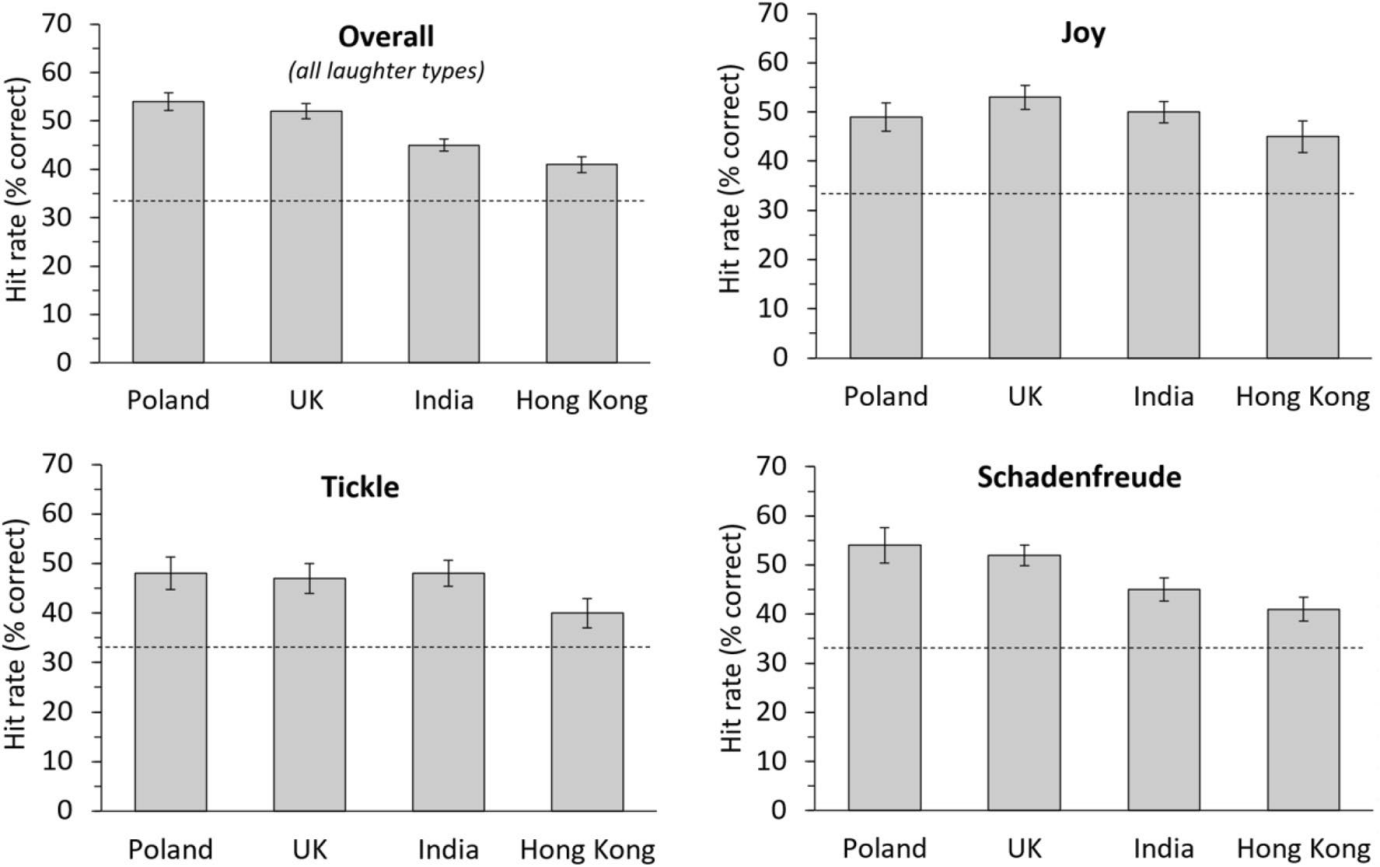
Recognition rates overall (across laughter types) and per laughter type, split by group. Horizontal dashed line indicates the guessing probability (chance level) of 33%. Error bars denote standard error of the mean (SEM).

Across laughter types, correct recognition rates (Fig. 1, Table 3) were around 50% (chance level 33%), with the highest rate for the *Other* group (52.6%) and the lowest rate for *Hong Kong* (42.5%). Split up for group and laughter type, the highest recognition rate was 55.9% for schadenfreude in the *Other* group, and the lowest rate was 40.1% for tickling in the *Hong Kong* group.

**Table 3:** Recognition rates (mean percent correct) for the three laughter types for the separate groups. Standard deviation and minimum and maximum in parentheses.

	Joy	Schadenfreude	Tickle	Overall
Poland	49.1 (12.8; 24–68)	53.8 (16.4; 24–86)	48.2 (14.8; 21–71)	49.9 (8.2; 33–61)
UK	52.9 (14.6; 28–84)	52.2 (12.7; 28–72)	47.2 (18.6; 24–81)	50.4 (9.6; 51–68)
Hong Kong	45.2 (14.5; 16–86)	41.4 (16.1; 10–62)	40.1 (17.5; 14–71)	42.5 (8.4; 28–61)
India	50.4 (16.2; 16–76)	45.1 (12.4; 14–83)	47.9 (14.7; 7–79)	48.2 (8.2; 31–64)
Other	53.3 (16.1; 22–84)	55.9 (13.9; 17–79)	49.6 (16; 10–81)	52.6 (8.2; 36–69)
Overall	50.6 (15; 16–86)	49.5 (15.2; 10–86)	47 (16.8; 7–81)	49 (9.1; 28–69)

Table 1 shows that the Hong Kong sample had a higher mean age (42.92 years) as compared to the other groups. To test weather this affected the results, we removed the 7 oldest participants, leaving a sample of 18 participants with a mean age of 33.44 years (SD 15.28, 18–59 years), which was comparable to the Polish sample. The recognition rates for this age-matched sample did not differ notably from the full sample. In more detail, the overall recognition rate across laughter types was 42.93% (N = 18) instead of 42.5% (N = 25), for Joy 43.89% (instead of 45.2% for N = 25), for Schadenfreude 41.76% (instead of 41.4% for N = 25), and for Tickle 42.59% (instead of 40.1% for N = 25). Therefore, the higher mean age of the Hong Kong sample did not affect the recognition rates.

### Can type of laughter be recognised within each group?

While for illustration the tables and figures show hit rates (percent correct), we used H_u_ and P_c_ for all statistical tests. All tests were based on the relative difference between H_u_ and P_c_ (i.e., H_u_ minus P_c_), reflecting recognition abilities. If H_u_ and P_c_ are identical, this difference is 0, and reflects that participants are not able to recognise laughter above chance level. In the following we call this measure H_u-pc_.

To test whether laughter can be recognised above chance level, we first calculated five (one for each culture group) one-sample t-tests of H_u-pc_ versus 0, averaged across all laughter types (Fig. 1 Panel Overall, Table 3). For all groups, recognition rates were significantly above the chance level of 33%. In more detail, overall recognition rate for Poland was 49.9% (one-sample t-test of H_u-pc_ vs. 0, t(19) = 8.944, *p* < 0.001, Cohen’s d = 4.104), for the UK 50.4% (t(36) = 10.199, *p* < 0.001, Cohen’s d = 3.400), for India 48.2% (t(44) = 10.978, *p* < 0.001, Cohen’s d = 3.310), for Hong Kong 42.5% (t(24) = 5.659, *p* < 0.001, Cohen’s d = 2.310).

Next, we tested whether each individual laughter type (i.e., joy, tickle, schadenfreude) can be recognised above chance level. For this, we calculated one-sample t-tests of H_u-pc_ versus 0 separate for each of the 5 groups and 3 laughter types (i.e., 15 tests in total). Results showed that within each group each laughter type was recognised significantly above chance level (all *p* < 0.001, except for Joy in Hong Kong, *p* = 0.0018, t-values 3.517 (Joy in *Hong Kong*) – 12.099 (Tickling in *Other*), df 24–44, see “supplementary online materials” for full statistics).

Above we calculated 20 t-tests overall, which inflates the likelihood of an alpha error (false positive). Accordingly, we controlled the family-wise error rate to *p* < 0.05 using Bonferroni correction (Bonferroni corrected critical *p* < 0.0025). For illustration, we applied the Bonferroni correction by multiplying the *p*-values of all the above analyses by 20 (capped at *p* = 1). Even after Bonferroni correction for 20 tests, all above results remain statistically significant. 17 of the 20 comparisons are *p* < 0.001 if Bonferroni corrected, only the individual laughter types for the *Hong Kong* group did not meet this level (Joy in Hong Kong *p* = 0.035, schadenfreude in Hong Kong *p* = 0.008, tickling in Hong Kong *p* = 0.001).

Recognition rates did not differ between male and female participants (Overall across all groups, mean females 49.18%, mean males 48.17%, independent-samples t-test t(159) = 1.182, *p* = 0.392). Data separate for each group are provided in the “Supplementary Online Materials”.

**Figure 2:**
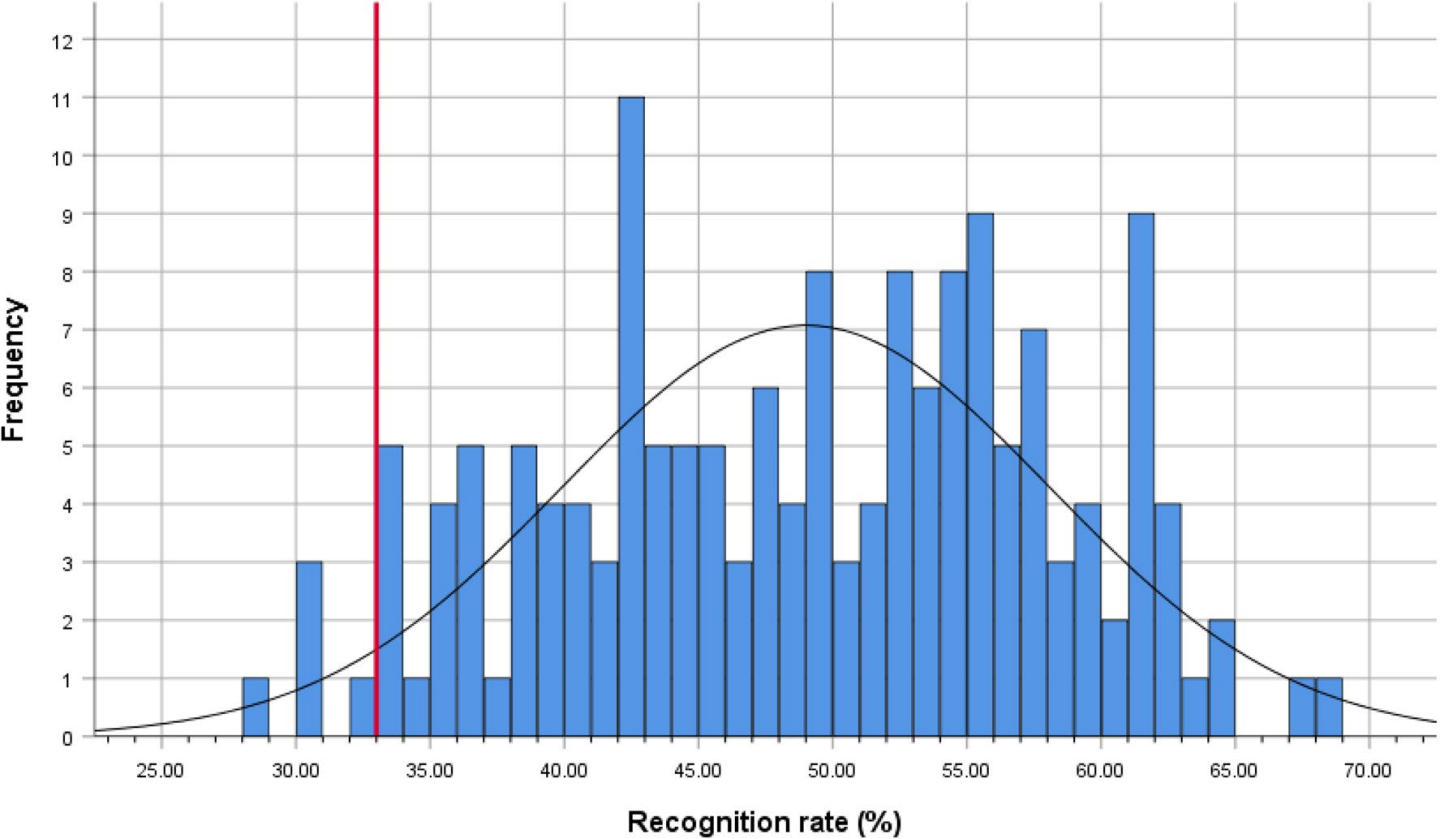
Histogram showing the overall recognition rates (averaged across all 3 laughter types) across all groups (N = 161). Bin width = 1%. The red vertical line shows the chance level of 33%. Separate histograms for each group can be found in the “Supplementary Online Materials”.

In the above analyses, we determined recognition accuracy across participants. Another view to inspect the data is to test how many participants individually show a significant recognition rate (cf. Fig. 2).

To test for statistical recognition in individual participants, we calculated a χ^2^-test for each participant, based on the expected and observed frequencies for Joy, Schadenfreude, and Tickling laughter. The χ^2^-tests revealed that 75% of the participants (121 out of 161 participants) showed significant recognition rates with *p* < 0.05 (χ^2^ > 5.991; 2 df), and 59% of the participants (95 out of 161) even at a significance threshold of *p* < 0.005 (χ^2^ > 10.597). Split by group, 85% of the Other participants (34 out of 29), 84% of the UK participants (31 out of 37), 80% of Indian participants (36 out of 45), 75% of Polish participants (15 out of 20), and 40% of participants from Hong Kong (10 out of 25) showed significant recognition rates with *p* < 0.05 (χ^2^ > 0.5991).

Taken together, these latter analyses and Fig. 2 show that the further above reported significant recognition rates on the group-level are not caused by a few ‘super-recognisers’, but instead reflect the majority of participants (97% of all participants with numerical scores above chance level; 75% statistically significant above chance level).

For a deeper understanding of how the different stimuli were classified into the different categories, we calculated the confusing matrix (Table 4). The confusion matrix shows that for each given stimulus category, the according response category was the most frequently chosen response. To identify potential confusions, we tested whether other categories beside the correct target category also showed significant hit rates above chance level of 33%. The only statistically significant confusion was that 36% of the Schadenfreude stimuli were classified as Joy, while the reverse relationship (Joy stimuli classified as Schadenfreude) was not significant. This confusion is expected to some extent, because Schadenfreude (literal translation from German would be harm-joy) per definition also contains an element of joy.

**Table 4:** Confusion matrix, average across all participants (N = 161).

Stimulus	Response		
	Joy	Schadenfreude	Tickle
Joy	51%***	34%	16%
Schadenfreude	36%*	49%***	15%
Tickle	30%	23%	47%***

Data in bold represent correct classification. Asterisks denote significant recognition above 33% chance level (*p < .05; **p < .01; ***p < .001; one-sample t-tests vs. 33%). Separate confusion matrices for each group can be found in the “Supplementary Online Materials”.

To assess whether participants (as ‘raters’) showed consistent patterns, we calculated intraclass correlation coefficients (ICCs) as a measure of interrater reliability (two-way mixed model; type consistency). Averaged across all groups, ICCs were high to very high (across all laughter types 0.841; Joy 0.797; Schadenfreude 0.774; Tickle 0.902). When split by group, the lower number of participants (raters) resulted in lower ICCs, which were roughly comparable across groups (averaged across the three laughter types, Poland 0.455; UK 0.523; India 0.622; Hong Kong 0.515; Other 0.507). The full data (split by group and laughter type and confidence intervals for all ICCs) can be found in the “Supplementary Online Materials”.

Taken together, the results show that all laughter types were significantly recognised above the chance level of 33%, with recognition rates varying between 40.1% (for Tickle laughter in the *Hong Kong* group) and 55.9% (for Schadenfreude in the *Other* group). Regarded on the level of individual participants, 97% of all participants showed recognition rates numerically above the chance level of 33%, and 75% of all participants showed statistically significant recognition.

### Do the recognition rates differ between groups?

Our second hypothesis was that the recognition rates might differ between groups, and that groups coming from more distant cultures may show lower recognition rates as compared to groups from closer cultures. Because Poland and UK, both European countries rather close to Germany, can be considered close cultures, and India and Hong Kong are both rather distant to Germany, we created two groups: *Close* (Poland, UK) and *Distant* (India, Hong Kong). The *Other* group was not considered for this analysis, because participants in that group came from all around the world.

The resulting *Close* group (N = 57) had an overall recognition rate of 52.2% (SD 8.56%), which was significantly different from the chance level of 33% (one-sample t-test of H_u-pc_ vs. 0, t(56) = 13.485, *p* < 0.001, Cohen’s d = 3.604). The *Distant* group (N = 70) had an overall recognition rate of 46.2% (SD 8.76%), which was significantly different from the chance level of 33% (one-sample t-test of H_u-pc_ vs. 0, t(69) = 11.735, *p* < 0.001, Cohen’s d = 2.825). In particular, the *Close* group had a 6% higher recognition rate as compared to the *Distant* group, which was significant (independent samples t-test of H_u-pc_ scores of the Close group vs. H_u-pc_ scores of the Distant group, t(125) = 3.141, *p* = 0.002, Cohen’s d = 0.562).

The same pattern was observed when the laughter types were analysed separately, i.e. all laughter types were recognised above chance level in *Close* as well as *Distant* groups (all *p* < 0.001), and recognition rates were always significantly higher in the *Close* as compared to the *Distant* group (Joy: Close 53.4%, Distant 48.5%, t(125) = 2.197, *p* = 0.030; Schadenfreude: Close 54.4%, Distant 43.7%, t(125) = 2.372, *p* = 0.019; Tickle: Close 49.2%, Distant 45.1%, t(125) = 2.977, *p* = 0.003).

To test whether laughter type and group may interact, we calculated a 2 × 3 mixed ANOVA with the between-subject factor Group (Close, Distant) and the within-subject factor Laughter Type (joy, schadenfreude, tickle). The dependent variable was H_u-pc_. Across the groups, the recognition rates differed significantly between laughter types (main effect Laughter Type, F(2, 250) = 84.552, *p* < 0.001, partial η^2^ = 0.403). Across laughter types, the recognition rates also differed significantly between groups (main effect Group, F(1, 125) = 9.864, *p* = 0.002, partial η^2^ = 0.073). The interaction between Laughter Type and Group only approached significance (F(2, 250) = 2.751, *p* = 0.066, partial η^2^ = 0.022).

## Discussion

### Summary of results

The first aim of the current study was to test whether the type of laughter (joy, schadenfreude, tickling) spontaneously produced by German speakers can be recognised by a large sample of listeners from different cultural backgrounds. Results showed that listeners from Poland, the UK, India, Hong Kong, and a mixed group of other cultures were all able to recognise each laughter type significantly above chance level, with recognition rates varying between 40.1% and 55.9% (chance level 33%). The second aim of the current study was to test whether the cultural background may modulate the recognition rates. We found that cultures close to Germany (Poland and UK) showed on average 6% higher recognition rates than the cultures distant to Germany (India and Hong Kong) suggesting cultural influences in identifying affective laughter types.

### Cross-cultural recognition of laughter

We found that laughter sounds can be classified according to the affective state of the sender significantly above chance level by listeners of diverse cultural backgrounds, suggesting that laughter expression is not culture specific. That affective laughter types can be identified cross-culturally is in line with findings that also other socially relevant information is communicated to outgroup members via laughter. For example, listeners of different cultures can tell if spontaneous laughs produced in conversations are uttered in company of a friend or of a romantic partner, even when the laugher was not from the listener’s culture^6^.

Moreover, Bryant et al.^5^ showed that listeners of 24 different societies were able to tell if laughter was directed to a friend or a stranger, irrespective of the cultural background of the laugher (with recognition rate of 53–67%; chance level 50%). Interestingly, listeners were able to classify laughter even if the laughter sound was very short, suggesting that classification of laughter affords a rapid appraisal of affiliation^5^.

In a study by Kamiloğlu and colleagues^25^, Japanese and Dutch listeners were even able to tell the cultural background of the laughter just by listening to different laughter sounds. In addition, there is increasing evidence that listeners can tell if the laughter was emitted spontaneously or if it was produced voluntarily (i.e. if somebody laughs pretending to enjoy a joke), again irrespective of the laugher’s culture^7,25,26^.

Interestingly, Sauter et al.^27^ investigated a diverse pool of different nonverbal vocalizations (such as laughter, screams, moans, cheers) and found that laughter was the only nonverbal vocalization that was reliably classified cross-culturally by listeners of distant cultures, namely Himba and English listeners. Moreover, both listener groups agreed that laughter communicates amusement and is uttered when being tickled. Our results show that also negative laughs, such as schadenfreude laughter, can be classified cross-culturally, and that listeners are able to distinguish between amusement laughter (“laughing with somebody”, in our study: joyful and tickling laughter) and negative laughter (“laughing at somebody”, in our study: schadenfreude laughter) irrespective of the cultural background. This suggests that while laughter has primarily evolved as a play signal between nonhuman primates that signals the enjoyment of physical play^27^, it can also be used to ridicule and exclude others from group context^32^. Importantly, our study suggests that such a negative message is also communicated reliably to outgroup members that are not part of the laugher’s culture.

### Cross-cultural differences

Research on the cross-cultural expression of emotions in the human voice in general has consistently demonstrated an in-group advantage, i.e. listeners from the same culture as the sender recognise emotions with higher accuracy than listeners from a different culture^24^. In addition, there appears to be a gradient so that the more distant sender’s and listener’s cultures are, the lower the emotional recognition tends to be^22,24^. Our findings are in line with this, as close cultures (Poland and UK) showed on average 6% higher recognition accuracy as compared to distant cultures (India and Hong Kong).

Our findings are in line with research showing that listeners performed better when classifying nonverbal vocalizations (such as laughter, screams, moans, cheers) produced by members of their own culture^27^. Interestingly, in order to show an advantage in classifying signals from ingroup members, it is not necessary to be able to consciously differentiate between ingroup and outgroup signals^28^, suggesting that affective appraisal is a very quick subconscious process.

In contrast to our results, Farley and colleagues^6^ found that, irrespective of the cultural background of the laugher, listeners are equally good at classifying laughter emitted by friends and romantic partners (recognition rate 62–69%; chance level 50%). The authors suggested that the lack of an ingroup advantage might be due to limitations in sample size or to having limited information about the cultural background of the listeners^6^. Therefore, whether appraisal of affiliation shows a similar ingroup effect as appraisal of the affective state is still unclear.

Overall, while it is still unclear whether listeners are able to identify the sender’s cultural background in laughter^25,but 26^, listeners of close cultures show an advantage when decoding information relevant for social cognition, such as affective content and maybe also group affiliation, even when listening to short segments of laughter.

### Communication of information in laughter

Laughter can be differentiated according to the sender’s affective state on the basis of the mere acoustic signal, regardless of whether it is produced spontaneously (see also^12^; recognition rates 35–39%; chance level 25%) or voluntarily (^13^ recognition rates 37–50%; chance level 25%). The current results confirm this for a cross-cultural participant sample.

For laughter produced by actors, we have shown that the laughter type is encoded in acoustic parameters, such as peak frequency and centre of gravity^33^, and the emotional dimensions arousal, dominance, and valence are encoded in fundamental frequency and temporal parameters^34^. That the acoustical signal of laughter alone can communicate information about the sender has also been reported by Wood and colleagues who found that the laugh’s acoustics depend on the affective context in which the laughter was emitted, i.e. reward, affiliation, and dominance, in spontaneous laughs^15^ as well as in voluntary laughs^14^. Likewise, it has been shown that mirthful laughs differ acoustically from polite laughs spontaneously produced in conversations^16^.

That the affective state of the sender is communicated by the mere acoustical signal has also been shown for other nonverbal vocalizations. For example, Holz and colleagues^35^ found that listeners are able to judge emotional expressions above chance level in voluntary nonverbal affective vocalizations, such as cries, moans, and screams (recognition rates 14–22%; chance level 17%). A similar study was done by Engelberg et al.^36^ who, similar to our studies on laughter, did not compare different non-verbal vocalisations, but investigated one single type of non-verbal vocalisation, i.e. screams. Engelberg et al.^36^ showed that the acoustical properties of screams emitted in various affective contexts taken from movies and TV presentations differed depending on the affective context. Therefore, growing evidence suggests that the affective state is not only communicated by varying the type of non-verbal communication^27^, but that even single types of non-verbal vocalizations can communicate the affective state in a fine grained fashion (e.g. amusement laughter vs. schadenfreude laughter; surprise scream vs. anger scream).

However, some studies did not observe that the mere acoustical signal of laughter can communicate affective states^18,19^. The reasons for these discrepant findings are not clear but might at least partially be caused by differences in the methodological design. For example, Rychlowska et al. created situational contexts to elicit certain emotions, such as eliciting schadenfreude by participants watching someone else losing a game. However, the situational contexts may have resulted in some participants experiencing other emotions, such as empathy or pity when watching someone losing. This incongruence between intended situational context and perceived emotion is something we observed in Szameitat et al.^12^, where for example by far not all participants reported having felt schadenfreude after watching videos which we deliberately selected to elicit schadenfreude. Noteworthy, in the current study each laughter stimulus is assigned to the respective category (e.g. joy or schadenfreude) based on the emotion the laugher (sender) reported, and not based on the nature of the video they watched (the tickling laughter was elicited by actual tickling among friends). Therefore, generally we believe that it is the actually experienced affective state which is encoded into the laughter signal, and not the situational context as such. Importantly, the same situational context may lead to very different affective responses, possibly preventing finding clear results.

### Limitations

The current study was not designed as a cross-cultural study. For example, the sample sizes of some groups are comparatively small for a cross-cultural study (e.g., Poland with N = 20). Therefore, one should be cautious in making strong inferences about specific characteristics of the cultural groups investigated. Given that there are only very few studies investigating the communication of affective state in laughter, we believe that the inclusion of a variety of different cultural groups had two main benefits. First, we provided the proof that, in the most generic sense, affective communication in laughter can work across cultural boundaries. While this has been shown for other aspects of laughter^5^, this is the first time that it is shown for the communication of the laugher’s affective state. Second, we were able to show that this communication appears to be modulated by the cultural distance. Again, caution may be advised when inferring about specific cultures, but the current study showed that, in general, affective communication in laughter aligns with the common patterns shown for other forms of affective communication.

While we assessed the cultural backgrounds of the participants and their parents, we did not assess whether participants had contact to the German culture. It could be argued that contact to the German culture, e.g. by visits to Germany or having friends from Germany, may affect the sensitivity to perceiving the correct affective state of German laughter. Future studies may test whether such factors may indeed affect the recognition rates.

### Conclusion

Overall, the mere acoustical signal of laughter is a tool to communicate different aspects relevant for social communication, such as affiliation between sender and listener^5,6^, honesty of the signal (spontaneous or voluntarily produced)^7,25^, and affective state of the sender, as shown in the current study and by others^12,14,15^. While we believe that the combination of vocal laughter expression, emotional facial expression, and knowledge of the situational context is most potent for the communication of affective social information, our key finding is that the acoustical signal alone is already sufficient to communicate affective information across a range of cultures.

### Supplementary Information

Supplementary Information.

## Data Availability

All data generated in this study are included in the supplementary online materials of this published article.
