# Supplementary material for: Recognition of emotions in German laughter across cultures
Source: Sci Rep. 2024 Feb 6;14:3052. doi: 10.1038/s41598-024-53646-4 (PMC10847427; doi:10.1038/s41598-024-53646-4)
Supplement: Supplementary file 1 — Supplementary Information. [file 41598_2024_53646_MOESM1_ESM.pdf]

Supplementary Online Materials

for the manuscript

Recognition of German laughter across cultures

Diana P. Szameitat and Andre J. Szameitat

Scientific Reports

Contents

1. Full statistics for “one-sample t-tests of H_{u-pc} versus 0 separate for each of the 5 groups and 3 emotions, i.e. 15 tests in total” in the Results section 3.2 2

2. Histograms for all groups 4

3. Confusion matrices for all groups 7

4. Recognition Rates split by Sex 8

5. Intraclass Correlation Coefficients by group and laughter type 9

1. Full statistics for "one-sample t-tests of $H_{u\text{-}pc}$ versus 0 separate for each of the 5 groups and 3 emotions, i.e. 15 tests in total" in the Results section 3.2

Sdf = Schadenfreude; Tic = Tickling. Bonferroni p-value correction for 20 tests (i.e., original p-value multiplied by 20).

One-Sample Test[a] POLAND

	Test Value = 0						
	t	df	Sig. (2-tailed)	Mean Difference	95% Confidence Interval of the Difference: Lower	Upper	Bonferroni p-value
Overall	8.944	19	0.0000000	0.15899	0.1218	0.1962	0.0000006
Joy	6.574	19	0.0000027	0.12564	0.0856	0.1656	0.0000542
Sdf	5.905	19	0.0000110	0.09057	0.0585	0.1227	0.0002199
Tic	9.026	19	0.0000000	0.26075	0.2003	0.3212	0.0000005

One-Sample Test[a] UK

	Test Value = 0						
	t	df	Sig. (2-tailed)	Mean Difference	95% Confidence Interval of the Difference: Lower	Upper	Bonferroni p-value
Overall	10.199	36	0.0000000	0.15874	0.1272	0.1903	0.0000000
Joy	7.492	36	0.0000000	0.12155	0.0886	0.1545	0.0000002
Sdf	8.224	36	0.0000000	0.11901	0.0897	0.1484	0.0000000
Tic	8.959	36	0.0000000	0.23565	0.1823	0.2890	0.0000000

One-Sample Test[a] INDIA

	Test Value = 0						
	t	df	Sig. (2-tailed)	Mean Difference	95% Confidence Interval of the Difference: Lower	Upper	Bonferroni p-value
Overall	10.978	44	0.0000000	0.13052	0.1066	0.1545	0.0000000
Joy	8.993	44	0.0000000	0.10863	0.0843	0.1330	0.0000000
Sdf	7.353	44	0.0000000	0.08062	0.0585	0.1027	0.0000001
Tic	10.899	44	0.0000000	0.20231	0.1649	0.2397	0.0000000

One-Sample Test[a] HONGKONG

	Test Value = 0						
	t	df	Sig. (2-tailed)	Mean Difference	95% Confidence Interval of the Difference: Lower	Upper	Bonferroni p-value
Overall	5.659	24	0.0000079	0.07805	0.0496	0.1065	0.0001584
Joy	3.517	24	0.0017646	0.05249	0.0217	0.0833	0.0352912
Sdf	4.112	24	0.0003973	0.06669	0.0332	0.1002	0.0079469
Tic	4.889	24	0.0000551	0.11498	0.0664	0.1635	0.0011026

One-Sample Test[a] OTHER							
	Test Value = 0						
	t	df	Sig. (2-tailed)	Mean Difference	95% Confidence Interval of the Difference		Bonferroni p-value
					Lower	Upper	
Overall	13.240	33	0.0000000	0.18099	0.1532	0.2088	0.0000000
Joy	7.484	33	0.0000000	0.12865	0.0937	0.1636	0.0000003
Sdf	9.906	33	0.0000000	0.14358	0.1141	0.1731	0.0000000
Tic	12.099	33	0.0000000	0.27075	0.2252	0.3163	0.0000000

2. Histograms for all groups

Below histograms show the overall recognition rates (averaged across all 3 laughter types) across all groups (panel A) and split for groups (N = 161). Bin width=1%. The red vertical line shows the chance level of 33%.

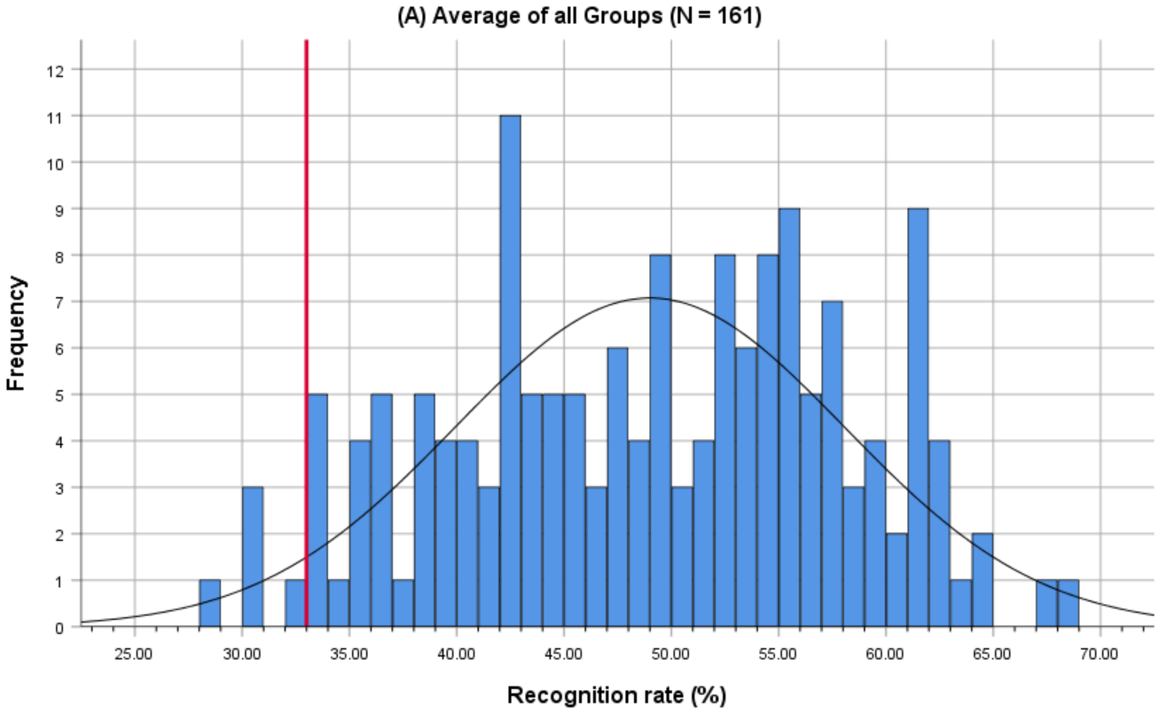

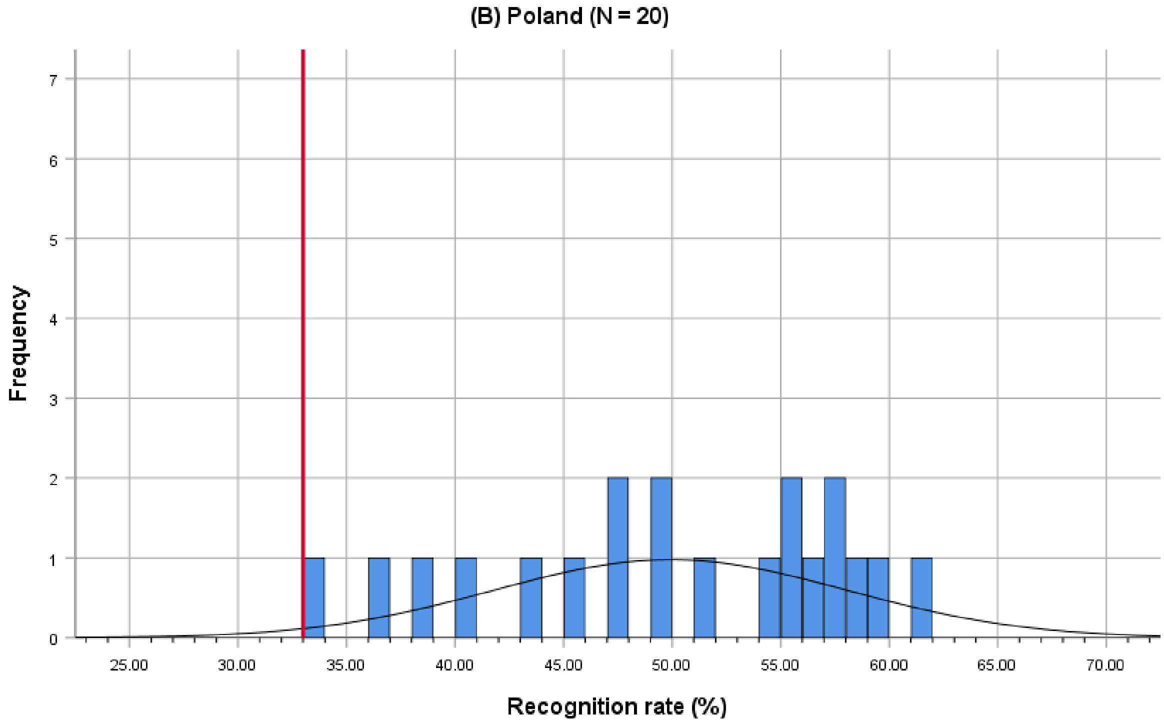

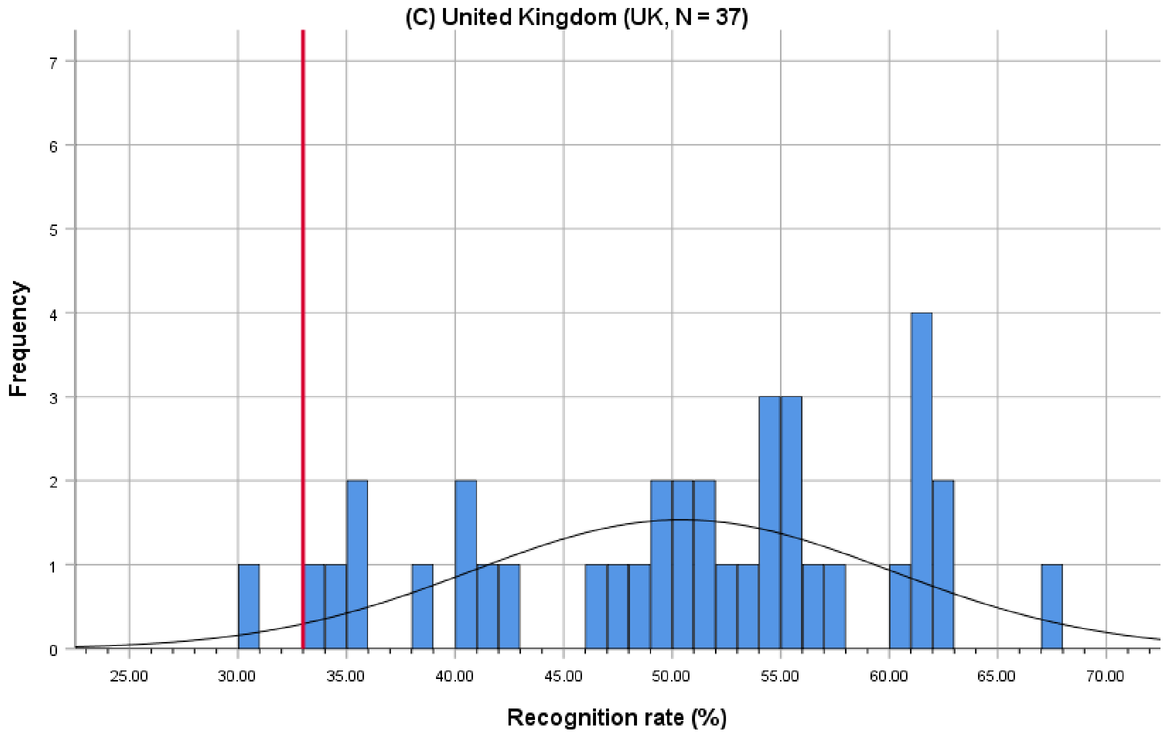
(C) United Kingdom (UK, N = 37)
Frequency
Recognition rate (%)
0
1
2
3
4
5
6
7
25.00
30.00
35.00
40.00
45.00
50.00
55.00
60.00
65.00
70.00

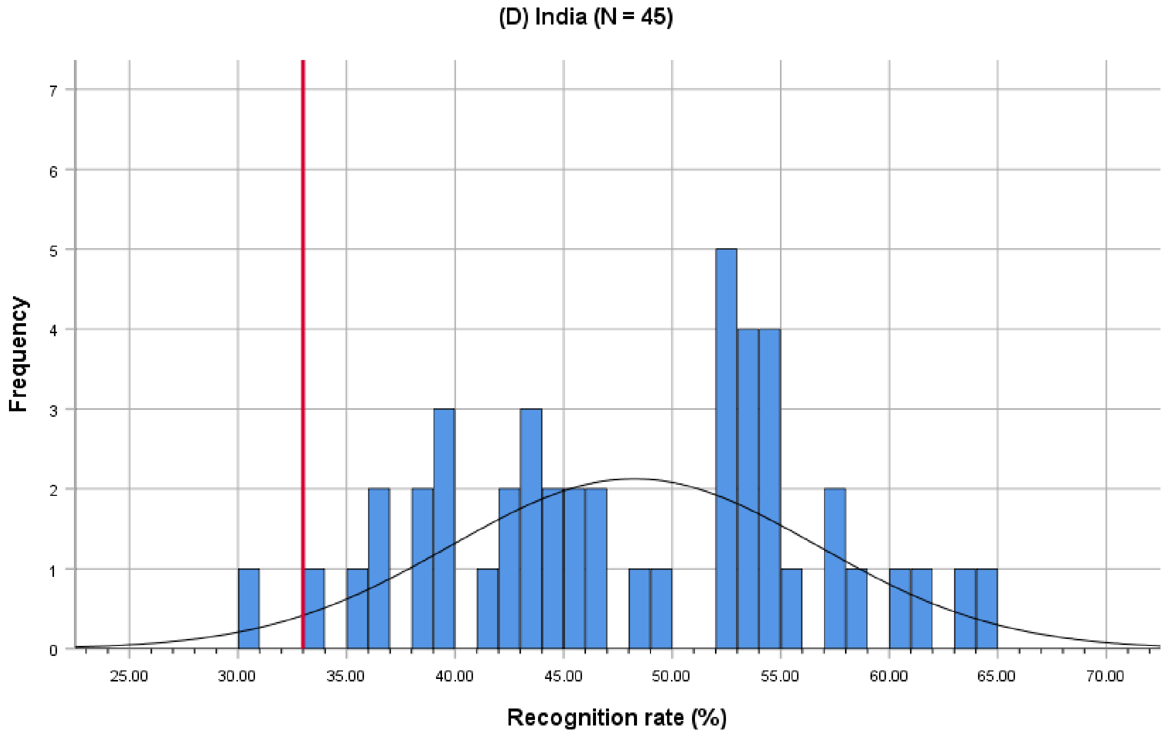
(D) India (N = 45)
Frequency
Recognition rate (%)
0
1
2
3
4
5
6
7
25.00
30.00
35.00
40.00
45.00
50.00
55.00
60.00
65.00
70.00

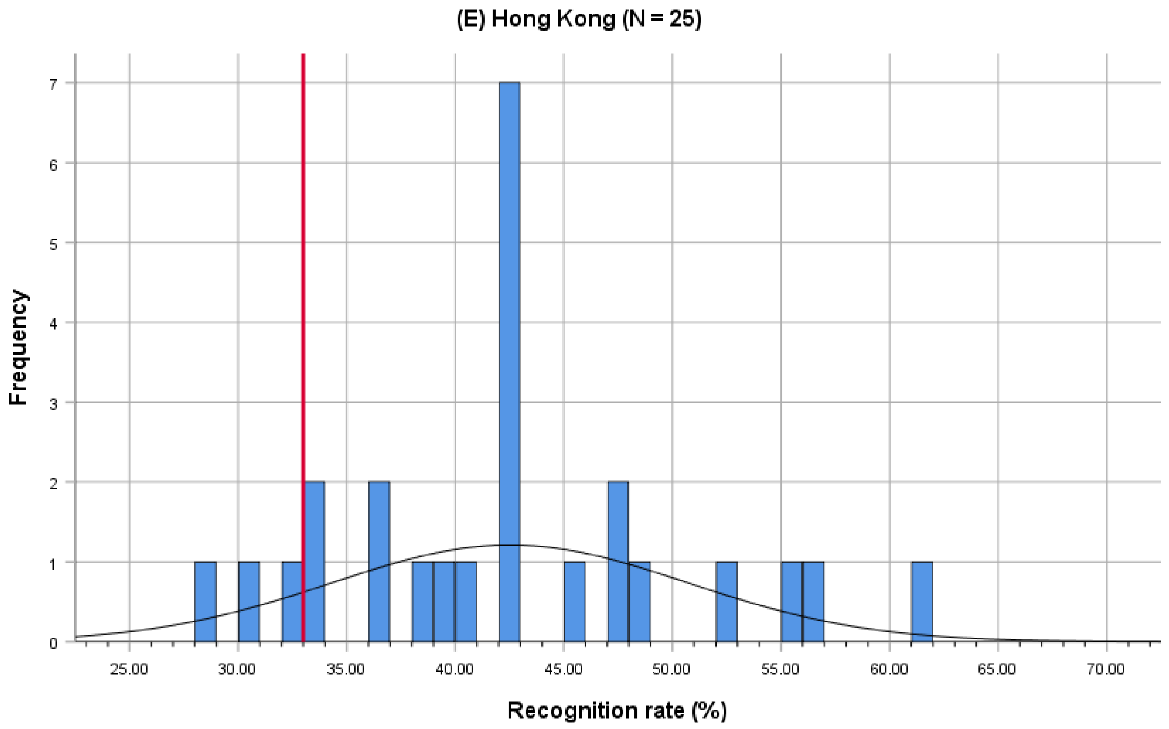
(E) Hong Kong (N = 25)
Frequency
Recognition rate (%)

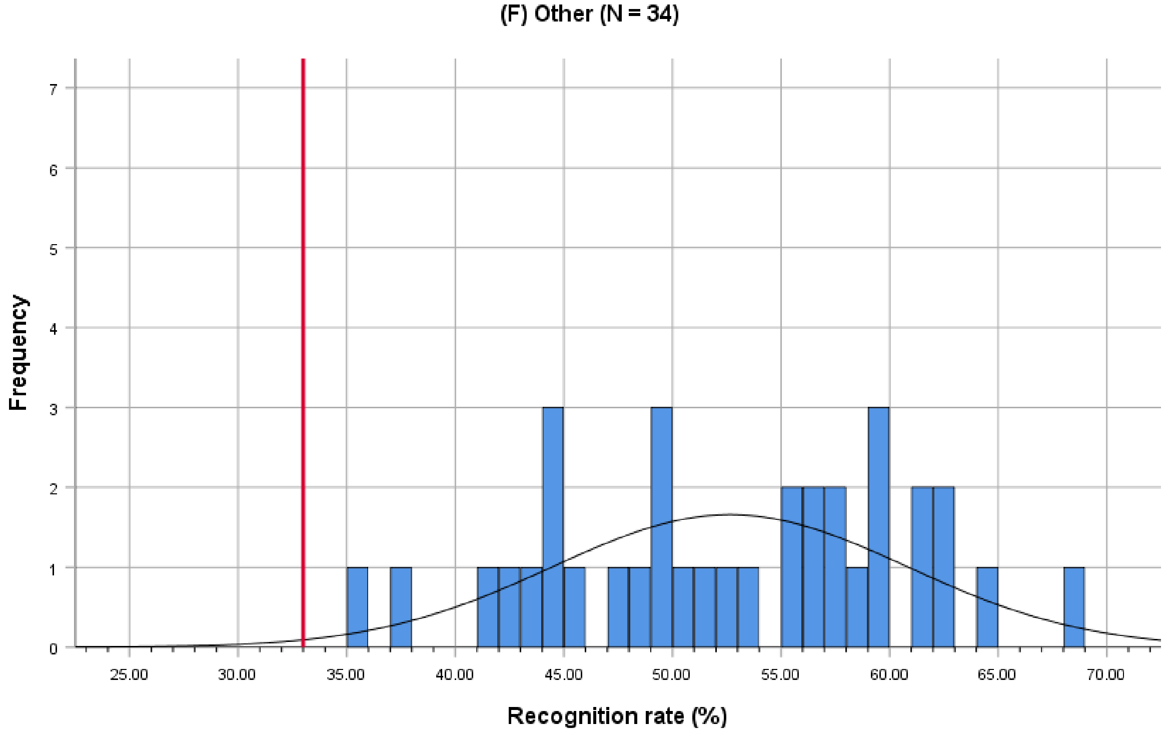
(F) Other (N = 34)
Frequency
Recognition rate (%)

3. Confusion matrices for all groups

Below are presented the confusion matrices, separate for each group. Data in bold represent correct classification. Asterisks denote significant recognition above 33% chance level (* p < .05; ** p < .01; *** p < .001; two-tailed one-sample t-tests vs 33%).

Group:	(All)	**Response**		
		Joy	Schadenfreude	Tickle
**Stimulus**	Joy	**51*** **	34	16
	Schadenfreude	36*	**49*** **	15
	Tickle	30	23	**47*** **

Group:	Poland	**Response**		
		Joy	Schadenfreude	Tickle
**Stimulus**	Joy	**49*** **	39*	12
	Schadenfreude	34	**54*** **	12
	Tickle	24	28	**48*** **

Group:	UK	**Response**		
		Joy	Schadenfreude	Tickle
**Stimulus**	Joy	**53*** **	34	14
	Schadenfreude	31	**53*** **	16
	Tickle	28	24	**49*** **

Group:	India	**Response**		
		Joy	Schadenfreude	Tickle
**Stimulus**	Joy	**51*** **	33	17
	Schadenfreude	37	**45** **	19
	Tickle	32	18	**50*** **

Group:	Hong Kong	**Response**		
		Joy	Schadenfreude	Tickle
**Stimulus**	Joy	**45*** **	32	23
	Schadenfreude	$38^{p=.07}$	**41** **	20
	Tickle	35	25	**40* **

Group:	Other	**Response**		
		Joy	Schadenfreude	Tickle
**Stimulus**	Joy	**48*** **	37	15
	Schadenfreude	31	**55*** **	14
	Tickle	30	19	**50*** **

4. Recognition Rates split by Sex

The table below shows the mean recognition rates (average across the 3 laughter types) and the results of independent-samples t-tests testing for sex differences:

	**Mean F (N)**	**Mean M (N)**	**T-test**	**p-value**
Overall	49.18% (83)	48.17% (78)	T(159) = 1.182	.392
Hong Kong	42.22% (15)	42.23% (10)	T(23) = .001	.999
India	50.22% (15)	46.57% (30)	T(43) = 1.407	.167
UK	52.89% (23)	47.22% (14)	T(35) = 1.888	.067
Other	51.33% (15)	54.21% (19)	T(32) = 1.086	.734
Poland	50.72% (15)	49.30% (5)	T(18) = .344	.735

5. Intraclass Correlation Coefficients by group and laughter type

We calculated the inter-rater reliability based on the correct (coded as 1) or incorrect (coded as 0) classification of each presented stimulus by each participant. To calculate the intraclass correlation coefficient (ICCs), we used a two-way mixed model and consistency as type. Below table shows the ICCs for averaged and split data (with the lower and upper bound confidence intervals (CI)).

Group	*Laughter type*	*ICC*	*CI-Lower*	*CI-Upper*
**All**	**ALL**	**.841**	**.798**	**.879**
All	Joy	.797	.708	.869
All	Schadenfreude	.774	.640	.876
All	Tickle	.902	.855	.940
**Poland**	**ALL**	**.455**	**.303**	**.586**
Poland	Joy	.458	.214	.654
Poland	Schadenfreude	.356	-.041	.651
Poland	Tickle	.551	.328	.726
**UK**	**ALL**	**.523**	**.393**	**.637**
UK	Joy	.478	.248	.665
UK	Schadenfreude	.328	-.076	.634
UK	Tickle	.666	.503	.795
**India**	**ALL**	**.622**	**.519**	**.712**
India	Joy	.508	.292	.684
India	Schadenfreude	.681	.490	.826
India	Tickle	.716	.578	.826
**Hong Kong**	**ALL**	**.515**	**.381**	**.631**
Hong Kong	Joy	.392	.121	.611
Hong Kong	Schadenfreude	.545	.268	.753
Hong Kong	Tickle	.634	.453	.776
**Other**	**ALL**	**.507**	**.372**	**.625**
Other	Joy	.465	.228	.657
Other	Schadenfreude	.357	-.032	.650
Other	Tickle	.633	.454	.775
